# A Mobile Internet Service for Self-Management of Physical Activity in People With Rheumatoid Arthritis: Challenges in Advancing the Co-Design Process During the Requirements Specification Phase

**DOI:** 10.2196/resprot.4824

**Published:** 2015-09-17

**Authors:** Åsa Revenäs, Cathrin Martin, Christina H. Opava, Maria Brusewitz, Christina Keller, Pernilla Åsenlöf

**Affiliations:** ^1^ Division of Physiotherapy Department of Neurobiology, Care Sciences and Society Karolinska Institutet Huddinge Sweden; ^2^ Division of Physiotherapy Department of Neuroscience Uppsala University Uppsala Sweden; ^3^ Department of Rheumatology Karolinska University Hospital Stockholm Sweden; ^4^ Swedish Rheumatism Association Stockholm Sweden; ^5^ Department of Informatics Jönköping International Business School Jönköping Sweden

**Keywords:** eHealth, participatory design, rheumatoid arthritis, user involvement, video observations

## Abstract

**Background:**

User involvement in the development of health care services is important for the viability, usability, and effectiveness of services. This study reports on the second step of the co-design process.

**Objective:**

The aim was to explore the significant challenges in advancing the co-design process during the requirements specification phase of a mobile Internet service for the self-management of physical activity (PA) in rheumatoid arthritis (RA).

**Methods:**

A participatory action research design was used to involve lead users and stakeholders as co-designers. Lead users (n=5), a clinical physiotherapist (n=1), researchers (n=2) with knowledge in PA in RA and behavioral learning theories, an eHealth strategist (n=1), and an officer from the patient organization (n=1) collaborated in 4 workshops. Data-collection methods included video recordings and naturalistic observations.

**Results:**

The inductive qualitative video-based analysis resulted in 1 overarching theme, merging perspectives, and 2 subthemes reflecting different aspects of merging: (1) finding a common starting point and (2) deciding on design solutions. Seven categories illustrated the specific challenges: reaching shared understanding of goals, clarifying and handling the complexity of participants’ roles, clarifying terminology related to system development, establishing the rationale for features, negotiating features, transforming ideas into concrete features, and participants’ alignment with the agreed goal and task.

**Conclusions:**

Co-designing the system requirements of a mobile Internet service including multiple stakeholders was a complex and extensive collaborative decision-making process. Considering, valuing, counterbalancing, and integrating different perspectives into agreements and solutions (ie, the merging of participants’ perspectives) were crucial for moving the process forward and were considered the core challenges of co-design. Further research is needed to replicate the results and to increase knowledge on key factors for a successful co-design of health care services.

## Introduction

### Background

User involvement in the development of health care services is important for the viability, usability, and effectiveness of services [1-3]. However, even when the users are involved, the development process may be unsuccessful, and the service may not be accepted and used. Previous research has reported diverse results regarding both benefits and drawbacks of user involvement [1,2]. One explanation for the drawbacks may be that little is known about the various aspects of collaboration associated with the development of a successful service [4,5].

Co-design implies active involvement of lead users (ie, potential users of the future service) to incorporate their experiences and knowledge into the new service [6]. “User-centered design” [7] and “participatory design” [1] are umbrella terms for design strategies. Experience-based design (EBD) [6] is a subform of participatory design that involves including lead users as co-designers. EBD uses principles from design science including architecture, product, and computer design to make the service or product safe, effective, and enjoyable for the user. Because the users’ experiences are essential to providing optimal care, they have been used to improve health care services [8]. EBD is also complementary with personalized care, which focuses on the individuals’ experiences, preferences, and goals in the provision of optimal care [9,10].

Co-design also denotes a collaboration between the stakeholders and system developers. Collaboration can be defined as the interaction between the stakeholders and system developers with the aim of achieving a shared goal [11]. Successful collaborations may be a key factor in the outcome of a project [12,13]. However, previous research related to co-design has primarily described the process on a macro level, for example, the benefits and drawbacks regarding money, time, and how user participation informs the new service [2,14]. Empirical studies describing the collaboration between co-design participants are scarce [2,4,5]. We were able to identify only 1 previous study that used video recordings to describe the collaboration during co-design meetings [15]. Therefore, research describing the collaboration during co-design is needed to extend our knowledge on effective collaborations to move the process forward.

### Study Objective and Overview

This study will provide a description of the challenges observed during co-design meetings. During the first step of the ongoing project, lead users presented ideas on core features that are to be included in the future service [16]. During the second step of the co-design, lead users, a clinical physiotherapist, researchers with knowledge in physical activity (PA) in rheumatoid arthritis (RA) and behavioral learning theory, an eHealth strategist, and an officer from the patient organization collaborated to provide data for the system requirements and specification of the future service [17]. The aim of this study was to explore the challenges in advancing the co-design process during the requirements specification phase of a mobile Internet service for the self-management of PA in RA.

## Methods

### Design

To explore the challenges in advancing the co-design process, an inductive, qualitative, participatory-action research design was applied [18]. The co-design process was performed during 4 workshops in February and March, 2013, at Uppsala University, Sweden.

Data collection included video recordings [19] and naturalistic observations [20]. The purpose was to capture situations and events in which the co-design participants discussed issues deemed important for advancing the co-design process. This study was approved by the regional ethical review board in Stockholm (D nr 2010/1101-31/5).

### Selection and Recruitment of Participants

The co-design group (n=10) was formed to create a feasible workgroup and to capture different perspectives (ie, experiential and theoretical knowledge). Potential co-design participants were identified through our research and clinical networks and were invited by email by the first author. The inclusion criteria were adequate Swedish communication skills and access to the Internet with confidence in using the Internet. Furthermore, the participants were chosen to include different perspectives, including experiential knowledge in living with RA, clinical experience in supporting individuals with RA to be physically active, theoretical knowledge on behavioral learning theory, evidence for PA in RA, and/or service design.

Potential co-design participants who provided preliminary consent were informed about the study by the first author. Written information and a questionnaire on background characteristics, expertise, PA behavior, and Internet habits were provided by mail or email. Participants provided their final consent for participation by attending the first workshop. All but 1 of the participants agreed to attend all 4 workshops.

The participants included 5 lead users, including a patient research partner, 2 researchers with knowledge in behavioral learning theories and PA in RA, 1 clinical physiotherapist, 1 officer from the Swedish Rheumatism Association, and 1 eHealth strategist. Three of the participants were men, and the median age was 55 years (age range 34-73 years). All but one of the participants possessed a university degree. The lead users were chosen to reflect diversity regarding age, sex, years since diagnosis, and PA habits. A few of the participants reported experiential and/or theoretical knowledge in more than 1 of the perspectives (ie, experiential knowledge in living with RA, knowledge of behavioral learning theories and evidence for PA in RA, clinical experience in supporting individuals with RA to adopt and maintain PA, and/or service design).

### Planning and Arranging the Co-Design Workshops

A pilot workshop was held before the start of the study to test the data-collection procedures; for example, technical solutions for the video recordings and the feasibility of an observation protocol used by the observers. This resulted in decisions on how to arrange the participants’ seating and where to place the microphone and camera. In addition, it was decided that a technician was needed to set up the camera before each workshop to integrate the video recordings with the interactive.

**Figure 1:**
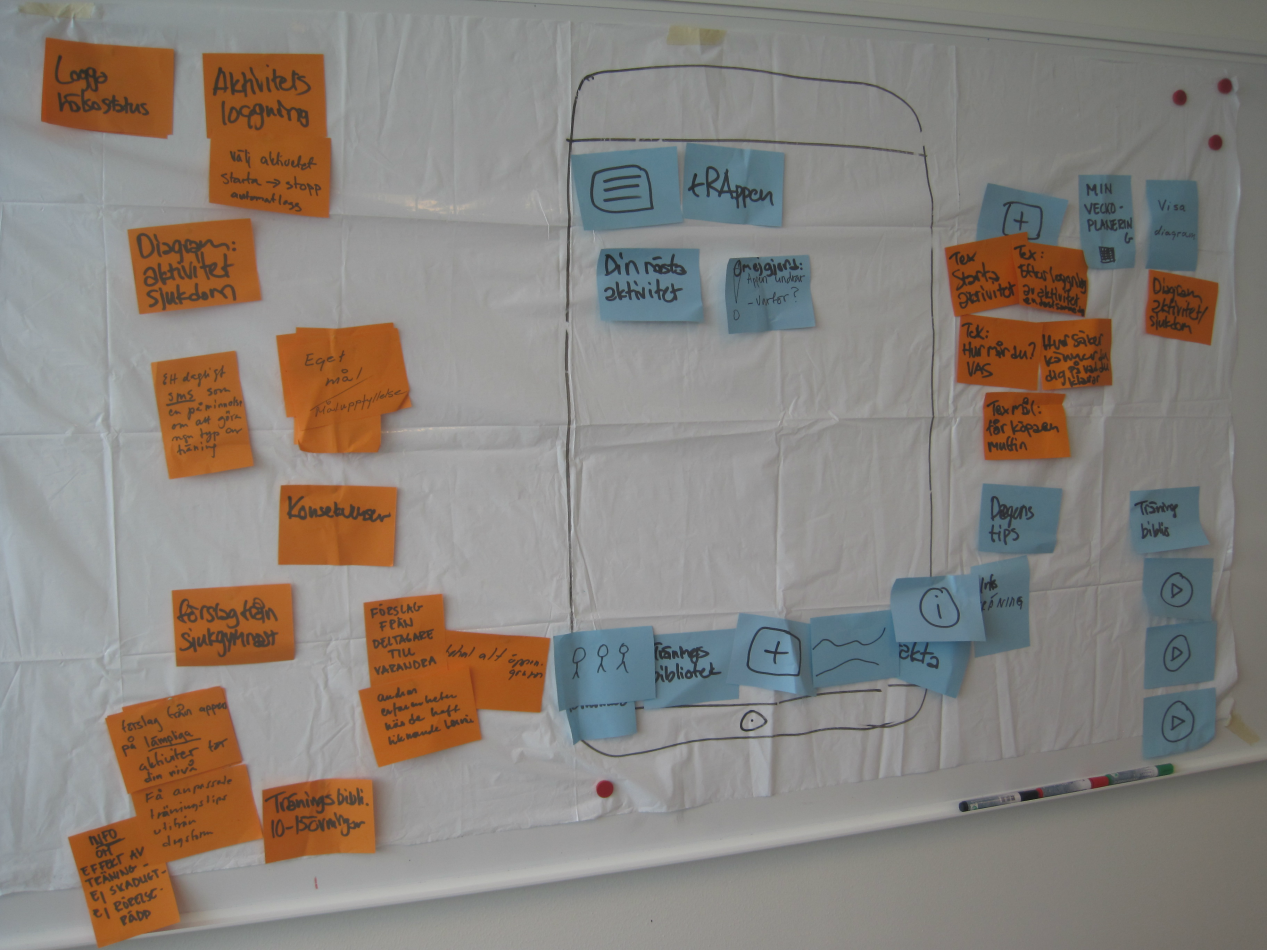
Plastic sheet with an outlined mobile phone.

The positioning of the camera can have a significant impact on the captured data and was carefully considered [19]. The camera was placed on a tripod and was positioned to capture the collaboration between participants, their faces and nonverbal actions, and references to the mediational means used (ie, interactive boards, an online notice board (Trello), and plastic sheets with an outlined mobile phone) (Figure 1). The interactive board and plastic sheet facilitated visualization of the future mobile Internet service during the discussion. They were also used to collect data on the system requirements and specification of the future service. These data were analyzed and presented elsewhere [17].

**Figure 2:**
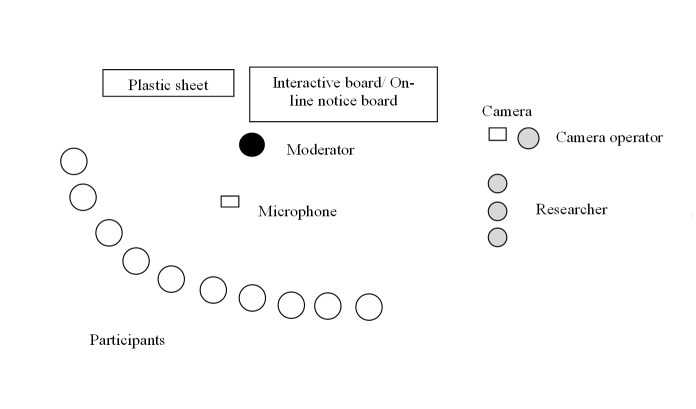
Arrangement of the co-design workshops.

To facilitate and collect data during the co-design workshops, 1 moderator and 3 researchers were present. The moderator, who had substantial experience with moderation, programming, and designing of digital devices, directed the workshops. The last author (PÅ, experienced in qualitative research and research on physiotherapy integrated with behavioral medicine) was responsible for alignment of the process. The camera operator (CM, experienced in qualitative research and video-based research) and 2 observers (ÅR, experienced in qualitative research and behavioral medicine; CK, experienced in qualitative research, naturalistic observations, and health informatics) collected data during the workshops. The observers only watched and took notes and did not participate in the workshops (Figure 2).

The 4 co-design workshops were performed at intervals of 1-4 weeks in university lecture rooms and lasted between 3½ and 5½ hours. The aim of the workshops was to provide basic data on the system requirements and specifications for the mobile Internet service. The first workshop started from the results of the first step of the co-design process: core features of a future Internet service as proposed by lead users participating in focus group interviews [16]. Thereafter, the first and last authors (ÅR and PÅ), the eHealth strategist, and the moderator planned the workshops iteratively; that is, each co-design workshop was built on the results and experiences from the previous workshop. Textbox 1 presents an overview of the major content and participants at each co-design workshop.

**Overview, major content, and attending participants at the co-design workshops.**

Workshop 1: Brainstorming

- Introduction
- Warm-up session
- Brainstorming on needs and proposed features

*Attending participants:* 3 lead users, 2 researchers, 1 physiotherapist (PT), 1 eHealth strategist, and 1 Swedish Rheumatism Association (SRA) officer

Workshop 2: Focusing

- Warm-up session
- Transforming needs to features
- Creation of the first prototype

*Attending participants:* 5 lead users, 2 researchers, 1 PT, 1 eHealth strategist, and 1 SRA officer

Workshop 3: Requirements specification

- Presentation of available physical activity apps
- Presentation of the first mobile phone prototype
- Creation of the second prototype

*Attending participants:* 4 lead users, 1 researcher, 1 PT, 1 eHealth strategist, and 1 SRA officer

Workshop 4: Requirements specification

- Presentation of the second mobile phone prototype
- Continuous specification of features

*Attending participants:* 4 lead users, 1 researcher, 1 PT, 1 eHealth strategist, and 1 SRA officer

### Data Collection

Data were collected by video recordings [19] and an observation protocol [20].

#### Video Recordings

A video camera (Sony PD170) was used to record the workshops. The camera was connected to a computer. The Wirecast program enabled integration of data from the camera and the interactive board. A conference microphone was used to assure good quality sound. The camera operator taped each workshop and saved the recordings on the computer every 20 minutes.

#### Observation Protocol

The 2 observers used an observation protocol, developed for this study, to take notes on contextual factors, the collaboration between participants, interesting situations and events, and the observers’ own reflections (ie, the atmosphere and feelings expressed during the workshops). These data were used as a complement to the video recordings and for identification of potential challenging events.

### Data Management and Analysis

The data consisted of approximately 16 hours of video recordings along with the observation protocols. The video recordings enabled repeated viewings of the relevant sequences where the challenges occurred. It also gave the other researchers the opportunity to discuss, confirm, reject, or adjust the analysis presented.

The inductive video-based analysis [19] was performed (ÅR) in 8 major steps (Textbox 2). The transfer of video data into text data resulted in a more thorough understanding of the challenges. A description of the transcript symbols and abbreviations used for participants are provided in Tables 1 and 2, respectively [21]. In addition, repetition of speech was reduced, and all the participants are referred to as female and the moderator as male.

**The 8 steps of the qualitative inductive video-based analysis.**

Step 1: Mapping of the co-design workshops

Between each workshop, the video recordings were described and classified according to the content in the workshops. During this preliminary viewing, notes were taken on situations that could be of interest after further analysis [19]. The 2 observation protocols were also compiled to obtain an overview of the observers’ notes.

Step 2: Familiarization

After the last workshop, the video recordings were viewed several times, and 107 sequences of interest were collected. The compiled observation protocols and the notes from the first step of analysis helped in identifying these situations.

Step 3: Building and rebuilding analytic collections

The identified sequences were viewed, compared, and labeled according to the participants’ actions. This step was iterative and involved labeling and relabeling of the sequences. The research questions were specified, which guided the viewing and resulted in modification and narrowing of the number of sequences. New sequences were added, and several sequences were lengthened, that is, 2 or more sequences became 1, were removed, or shortened. A total of 68 sequences remained.

Step 4: Categorization

Patterns began to emerge. Categories and subcategories were created and labeled. Selected sequences were further modified, and some were combined with other sequences.

Step 5: Representation

The sequences within each subcategory were viewed and prioritized according to how well they illustrated the challenge. In each subcategory, 1 or 2 sequences that were considered most illustrative were transcribed by the first author and finally translated into English.

Step 6: Formulating representation

During this stage, categories and subcategories were described in text, and the essence of each category was formulated. This resulted in further revision of the labeling and succession of the categories and subcategories. Themes were shaped.

Step 7: Validation

The notes from mapping and the observation protocols were checked again against the identified categories to make sure that no issues had been missed that could further answer the research questions. This resulted in the creation of 1 complementary category and modification of one of the subcategories.

Step 8: Refinement of results

The final step consisted of adjusting the theme, subthemes, categories, subcategories, and illustrations, and refining the labeling to enhance the text presentation of the challenges.

**Table 1:** Transcription symbols.^a^

Transcription symbol	Definition
[	Separate left double-row brackets indicate a point of overlapping onset
Bold letters	Words written in bold letters indicate some sort of stress or emphasis
Capital letters	Words written in capital letters indicate shouting
((M looks at RA))	Italic words or sentences in double parentheses are used to mark the transcriber’s descriptions of nonverbal signs and events
//	Two parallel lines indicate that the transcript has been shortened, that is, lines have been removed
=	Equal signs are used in pairs and indicate where a sentence stops and where it continues

^a^Modified according to Ten Have (1999) [21].

**Table 2:** Abbreviations used for the participants.

Abbreviation	Definition
RA	Lead users
RE	Researchers
PT	Physiotherapist
SRA	Officer from the Swedish Rheumatism Association
E	eHealth strategist
M	Moderator

Researcher triangulation was used to ensure trustworthiness during the analytic process. The first author participated in regular meetings with the second author, who guided the first author in the video-based analysis. Discussions involved deciphering what occurred during the selected sequences, including the challenges and their consequences for the process. Furthermore, labeling the theme, subthemes, categories, and subcategories evolved. The categorization and illustration of issues were discussed with the last author 3 times during the analysis process, which resulted in a consensus of the categorization and further refinement of the aim.

## Results

### Themes and Subthemes Identified

**Figure 3:**
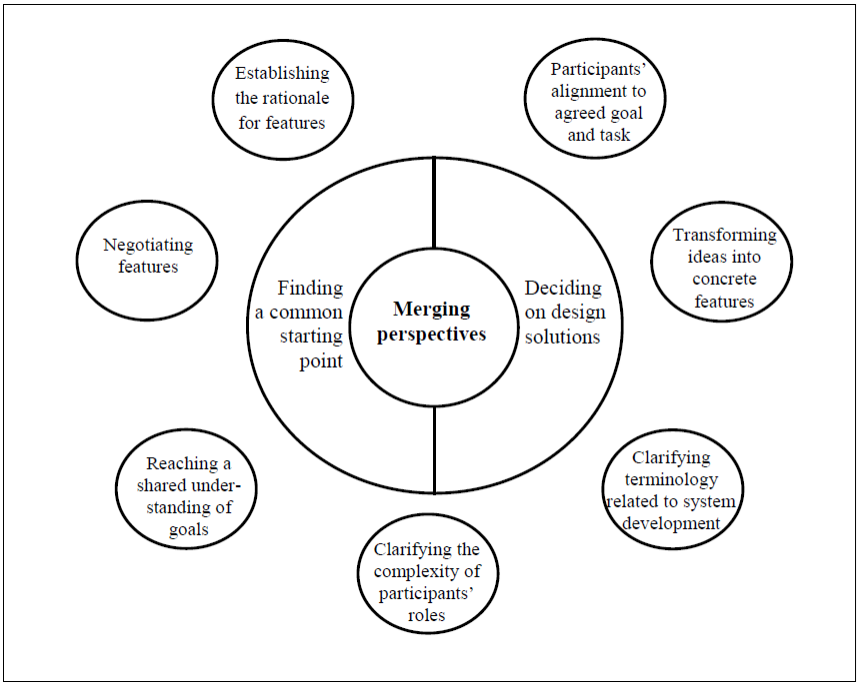
Overview of the overarching theme, subthemes, and categories describing the challenges in co-design.

The analysis resulted in 1 overarching theme and 2 subthemes. A total of 7 categories and 12 subcategories illustrated the challenges deemed important for advancing the co-design process toward the goal (Figure 3 and Table 3). The results will be outlined by descriptions of the content of the categories and subcategories. To further illustrate the challenges, excerpts of the transcripts will also be presented.

### Theme: Merging of Perspectives

The participants struggled to merge their individual perspectives during all phases of the co-design process. The participants shared, argued, and considered their different viewpoints, and integrated and counterbalanced these differences to find mutual agreements and solutions. In addition, 2 essential areas of merging were identified: “Finding a common starting point” and “Deciding on design solutions” (Figure 3).

**Table 3:** Overview of categories, subcategories, and specific illustrations of challenges in co-design.

Categories	Subcategories	Illustrations of challenges
Establishing the rationale for features	Combining different points of view	To imagine what feedback on personal progression means to the users; feedback on behavior goal achievement (physical activity, PA) or feedback on physical outcomes (eg, improved strength or mobility).
		To convey that exercise peers are important not only for inspiration and advice on new exercises but also for maintenance of PA.
		To acknowledge that home exercises for flexibility, as well as PA according to the recommendations, are health enhancing.
		To acknowledge both short-term symptom relief and long-term sustained health as a goal of PA.
		To acknowledge the importance of a personal, realistic PA maintenance goal, as well as one for pain relief.
	Identifying the significance of condition-specific characteristics on feature design	To choose how to provide feedback on health outcomes and what outcome measures to use.
		To understand whether it is possible to experience 100% health with rheumatoid arthritis (RA) and the consequences of designing graphs to monitor health reported using visual analog scales.
		To agree on how to present information on the negative consequences of sedentary life versus the positive consequences of a physically active life style.
		To decide on a feature to adjust PA goals to facilitate maintenance during exacerbation of RA.
Negotiating features	Consensus solutions emanating from different points of view	To decide on the scope of PA monitoring in the future service; should perform PA only according to established recommendations.
		To agree on the relationship between health and PA behavior and whether or not this is relevant to present in monitoring graphs.
	Finding necessary solutions for features despite remaining disagreements	To agree on whether or not to provide individualized advice, and if so, how? From the system, physiotherapist, or peers.
		To decide whether to include a video library with general exercise programs or not.
Reaching a shared understanding of goals of future service	Agree on overall aim of the mobile Internet service	To agree on a type of service to develop: a question-answer service or a service for PA behavioral change and maintenance.
	Agree on profile of target users	To choose whom to focus on: adopters or maintainers of PA, those wanting inspiration, or those in need of specific exercise advice.
		To agree on whether the target users should be described as just being curious about PA or already having an established interest in PA.
		To choose if the target users should have established contacts with health care or not.
Clarifying and handling the complexity of participants’ roles	Handling multiple roles	To imagine the users’ needs and consider those needs as well as their own needs as an academic or a professional need.
		To clarify what opinion to express; my personal opinion or the evidence-based opinion.
	Ensure all perspectives are voiced	To know when and how to synthesize arguments or solutions based on the perspectives of absent participants.
Clarifying terminology related to system development	Make sense of the terminology	To conceptualize and distinguish between needs and features.
		To make sense of the concept “target user group”; does it mean that some individuals are excluded or not?
Transforming ideas into concrete features	Recognizing needs and their corresponding features	To identify which features can encourage PA during periods with less motivation.
		To identify how users can get access to information when in need of encouragement.
		To identify features indicating when exercise has not been performed as planned.
	Visualizing the features on the mobile Internet service	To arrange the feature on a dummy.
		To imagine the experience of the welcome screen depending on preferred focus; peer group or self-monitoring.
Participants’ alignment to the agreed goal and task	Optimizing the mobile Internet service	To prioritize the most important features out of all suggested.
		To avoid a too complex service by restricting the number of features.
		To determine the correspondence between suggested features and the overall goal of the future service.

### Category: Establishing the Rationale for Features

#### Overview

The participants needed to combine their different points of view on issues related to PA in RA to be able to agree on appropriate features. They also needed to identify specific disease characteristics. The participants argued for their points of view, listened, and asked each other questions.

#### Combining Different Points of Views

Participants struggled to merge their different perspectives: for example, on feedback on personal progression and the role of peers in facilitating PA.

**Figure 4:**
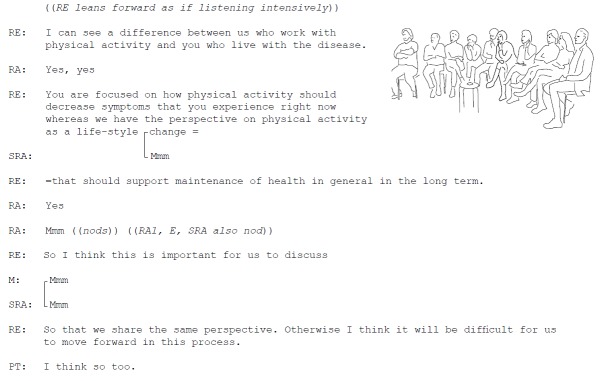
Excerpt 1. Different point of views.

The participants also had different views on the aim of PA itself, which affected discussions about which features to include in the future service. Was short-term symptom relief to be expected, or was the overall goal of PA general health enhancement? During the second workshop, one of the researchers raised these diverse perspectives (Excerpt 1; Figure 4).

The discussion ended with agreement on 2 main features of the future service: (1) peer support for specific advice and inspiration and (2) self-monitoring for personal goal setting, activity planning, and feedback on performance to support maintenance of PA.

#### Identifying the Significance of Condition-Specific Characteristics on Feature Design

One challenge was to agree on how specific characteristics connected to RA would impact feature design. For instance, whether it is possible for a person with RA to feel 100% healthy should be considered in the design of the anchors of graphs that monitor health. The disease course in RA was also considered because it is characterized by episodes of flares and remissions, which affect physical performance. This concern influenced the decision on whether to include a feature to easily review personal goals.

### Category: Negotiating Features

#### Overview

A salient challenge for the co-design participants was negotiation. The negotiations were observed as a continuous process between participants’ arguments before final solutions were agreed upon. Two different methods of reaching agreements were observed: consensus solutions emanating from different points of view and necessary solutions to features despite remaining disagreements.

#### Consensus Solutions Emanating From Different Points of View

The co-design process required participants to create solutions regarding which features to include in the future mobile Internet service even though they possessed different points of view. This way of negotiating was characterized by the participants’ ability to reach a consensus solution. This occurred when participants discussed what physical activities should be monitored in the future service and the relevance of displaying PA performance and general health perception in graphs. During the discussion of the latter, the moderator reflected over what he heard, “It sounds as if quite a lot of you with RA want to use graphs to monitor health as an excuse for NOT exercising. Is that not exactly the opposite of what we want the mobile Internet service to do?” The participants laughed and agreed.

#### Finding Necessary Solutions for Features Despite Remaining Disagreements

**Figure 5:**
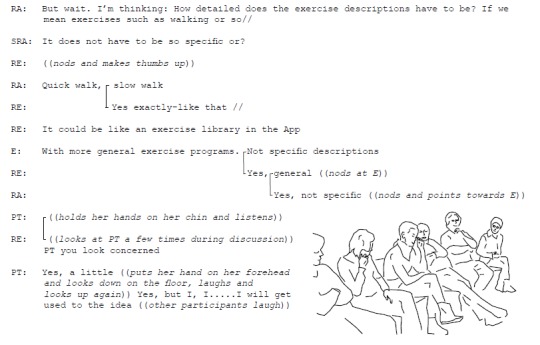
Excerpt 2. Finding necessary solutions for features despite remaining disagreements.

Even more challenging was the need to find a solution without consensus. The negotiation then ended with one of the participants giving up her opinion in favor of the opinion of the majority of the group. For example, this occurred when the participants agreed on a solution regarding if and how individualized advice should be provided in the future service and whether a video library should be included. This is illustrated by a discussion during Workshop 3 in Excerpt 2 (Figure 5).

### Category: Reaching a Shared Understanding of the Goals of the Future Service

#### Overview

A challenge faced in the initial stage of the co-design process was agreement of the participants on the overall aim of the future service and identification of the target users. The participants expressed frustration and uncertainty in not knowing which services to develop. The moderator repeatedly clarified that this was part of the process, and they all needed to agree to be able to specify the features in the future service.

#### Agree on Overall Aim of the Future Mobile Internet Service

**Figure 6:**
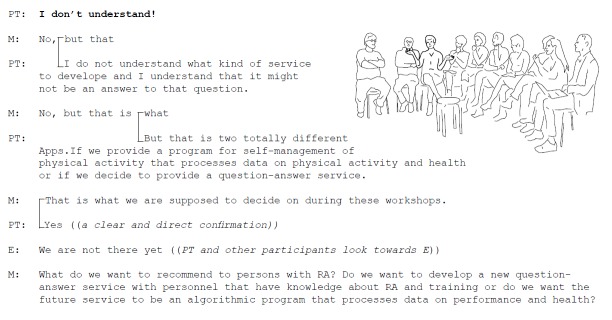
Excerpt 3. Agree on overall aim of the future mobile Internet service.

The future service should be designed to facilitate and inspire PA in people with RA. Participants discussed whether the service should be a question-answer service or a self-management service for PA behavior change and maintenance. Should the future service focus on which exercises to perform or include measures to facilitate PA behavior? Excerpt 3 exemplifies one of the discussions during Workshop 2 (Figure 6).

The discussion finally ended with an agreement that the mobile Internet service should serve as a self-management tool for PA behavior change and maintenance.

#### Agree on the Profile of Target Users

Initially, there was a need to agree on the characteristics of the users of the future service. For example, should the target users be new adopters or maintainers of PA? Was it sufficient to be just curious about PA or already have an established interest and ambition to be physically active? Should it be necessary to have established contacts with health care to obtain access to the service?

During Workshop 1, the participants discussed the characteristics of the target users. Ideas written on notes from each participant were posted on the wall, discussed, and voted on. The discussion finally led to an agreement for the target user group to be adults with RA who had some experience with and were prepared to self-manage PA. They should also be experienced Internet users.

### Category: Clarifying and Handling the Complexity of Participants’ Roles

#### Overview

Another challenge was to clarify the roles of the participants: for example, who they represented and what responsibilities they had. The participants needed to attend to their roles during the co-design workshops and they were also uncertain whose opinion to express. The moderator helped clarify their roles and ensured that different perspectives were explored.

#### Handling Multiple Roles

**Figure 7:**
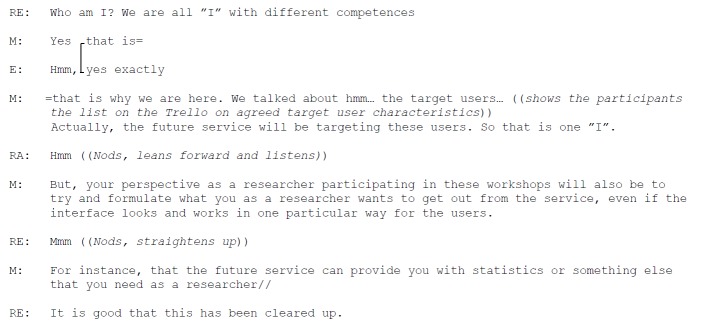
Excerpt 4. Handling multiple roles.

The academics and professionals encountered a challenge in determining the needs of the target users of the future service while satisfying their own needs as researchers, clinical physiotherapists, or eHealth strategists and in providing knowledge in their various areas of expertise. During Workshop 2, the moderator explained the reason for the discussion: the participants should have the “I perspective.” All the participants were present as experts who need to design a service that the expert group believed was optimal to inspire people with RA to live a physically active life. One of the researchers interrupted and requested further clarification on her roles (Excerpt 4; Figure 7).

The moderator continued and explained that all participants with an academic or professional role had to formulate what was important from their professional perspective. The moderator clarified the roles of the group members several times during the workshops.

#### Ensure All Perspectives Are Voiced

The participants tried to voice others’ perspectives if someone was absent but expressed difficulties in knowing when to do it and whose responsibility it was to ensure that all perspectives were heard. For instance, when one of the researchers was absent during one of the workshops due to illness, the other researcher suggested a feature that she believed would be important from a behavioral medicine perspective (a visual analog scale to measure a person’s self-efficacy or confidence in performing physical activities).

### Category: Clarifying Terminology Related to System Development

#### Overview

Because some of the participants had little or no experience in system development, it was challenging to conceptualize and understand the basic terminology used within this area. The moderator and participants with earlier experiences in system development repeatedly clarified and explained terms to enable discussions on how the future service should function.

#### Making Sense of Terminology

Participants had particular difficulties in distinguishing between the terms “needs” and “features.” *Needs* were defined as something that is needed to reach your goal and a *feature* was defined as something that fulfilled that need, that is, something that would be accomplished by use of the mobile Internet service. These definitions were needed for the participants to visualize their needs for the future service.

### Category: Transforming Ideas Into Concrete Features

#### Overview

The moderator had a difficult and strenuous task of helping participants recognize needs, transform those needs into features, and visualize the future service. He used prompts, such as scenarios and questions. He also used mediational means, such as a plastic sheet with notes, interactive boards, and programed prototypes to visualize the future service. However, there was also extensive collaboration between the participants and the moderator to specify and visualize features.

#### Recognizing Needs and Their Corresponding Features

First, it was crucial to identify which needs could be satisfied by specific features in the future service. For example, when one of the lead users expressed the need for encouragement when she felt less motivated to be physically active, the moderator asked, “Have you got any suggestions for a feature that could satisfy that need?” The moderator also described the scenario, “You have a need for information and you need someone to tell you why you should exercise. What feature could that correspond to?” Sometimes, the participants identified and expressed needs and transformed the needs into features. For example, 1 participant expressed the need for a consequence when she did not perform an exercise as planned. Another participant suggested that this could be satisfied by receiving feedback on the percentage of performed exercises compared with planned exercises.

#### Visualizing the Features on the Mobile Internet Service

A part of the co-design process was to visualize the future service. The participants arranged notes illustrating the buttons on a mobile phone on a plastic sheet attached to the wall. The moderator also used the interactive board to draw buttons according to participants’ suggestions. By changing the place and size of the outlined buttons, the participants could visualize how the design of the welcome screen influenced the first impression of the future service. In the later phases of the co-design process, prototypes of programmed mobile phone services were presented.

### Category: Participants’ Alignment to the Agreed Goal and Task

#### Overview

Another strenuous task for the moderator was to keep the participants aligned with a common goal during the co-design process to optimize the future service. He used prompts, summarized the discussions, and asked questions to guide the participants toward the task and goal achievements. The participants, by contrast, sometimes had difficulties in understanding why they needed to prioritize.

#### Optimizing the Mobile Internet Service

The most important features of the service needed to be prioritized, which caused some frustration. The future service needed to include desirable features while maintaining simplicity. An alignment between suggested features and the comprehensive goal of the service also needed to be secured. This was discussed during Workshop 3.

**Figure 8:**
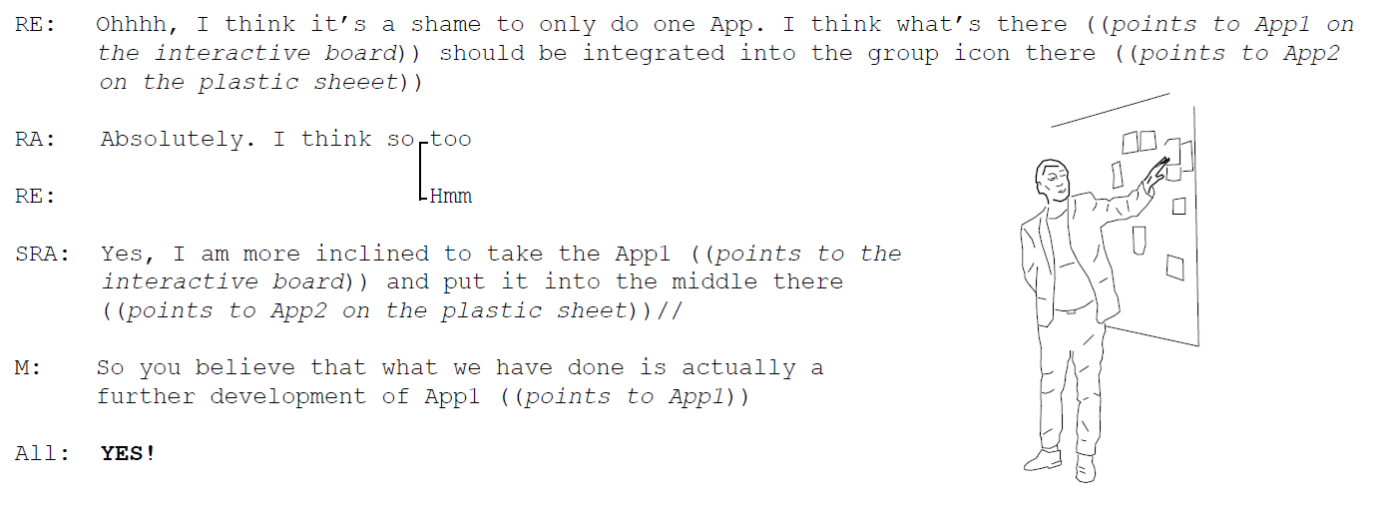
Excerpt 5. Optimizing the mobile Internet service.

The moderator summarized the features included in the 2 proposed prototypes of the future service. One prototype focused on peer support (app 1), and 1 prototype focused on self-monitoring (app 2). Did these 2 prototypes correspond to the overall aim of the future service (ie, to support self-management of PA in RA)? (Excerpt 5; Figure 8).

The participants determined that both peer support and self-monitoring were important and should be included but might be too complex for 1 service.

## Discussion

### Principal Findings

In this study, we learned that co-design is a complex and demanding process that faces several challenges. The challenges observed were related to reaching agreements and making decisions necessary to advance the co-design process. The merging of participants’ different perspectives was particularly challenging, yet it seemed to be the core activity in a successful co-design process.

The constant need to merge participants’ respective perspectives placed extensive demands on collaboration. Merging of perspectives can be described as the participants’ efforts to consider and value their different viewpoints and to integrate and counterbalance these into a mutual agreement to progress the co-design process toward the overall goal. The merging was built on individual perspectives, that is, experiential and theoretical knowledge and participants’ ability to share these with others. Merging also included finding solutions. Data showed that the co-design group exhibited 2 methods of negotiating and finding solutions: consensus solutions or solutions despite remaining disagreements. This description of the co-design group’s ability to merge is in accordance with what theoretically has been described as a “collectivity-of-practice” [22].

According to our results, the participants’ different points of view caused the process to be difficult and time consuming, which may lead to conflicts between lead users and system developers and negatively impact the process [23,24]. Relationship conflicts have a negative impact, whereas task conflicts improve group performance [25]. Our data indicated that the co-design group primarily experienced task conflicts. During these task conflicts, the participants presented, valued, and argued for their various perspectives, which widened the group’s decision basis. High demands were placed on the participants’ ability to merge their perspectives. Our assumption is that a health care service based on merged perspectives would be more feasible to both lead users and health care providers compared with services developed by more conventional means. However, this assumption must be studied further.

### Challenges Faced

One of the goals for the co-design process was to determine what type of service to recommend for people with RA in order to self-manage PA. The frame, structure, and features to include in the future service were not predetermined, which was frustrating and difficult for the participants. Consistent with previous research, stating clear goals contribute to a more effective collaboration in this study [26]. To handle this challenge, the future target users, the scope, and the aims of the service should be clarified early in the process.

Another challenge in the initial stage of co-design concerned the participants’ roles and responsibilities. The participants were unsure about their respective roles and expectations, which have previously been reported to negatively impact co-design performance [23,27]. Particularly, the academics and professionals had complex roles and were expected to contribute using their unique expertise while considering how to use their competencies without dominating. They were also expected to imagine the needs of the future users. The complex roles for researchers were previously described as a substantial challenge in participatory action research projects [28]. Our study expands that challenge to include not only researchers, but also the other participants in the co-design group. However, the researchers still expressed more concerns regarding their roles and the risk of dominance compared with other participants.

Another challenge concerned the recognition and transformation of the participants’ needs and ideas into concrete features to optimize the future service. The moderator, who had substantial experience with programming and designing digital devices, had a distinct role in this process. Needs and features were defined in terms that enabled the participants with no experience in this field to discuss the features on the future service. The moderator also programmed prototypes that allowed the participants to visualize the future service. The inclusion of engineers to help transform users’ needs into technical applications has been reported previously [27]. Our study emphasizes the importance of including experts in programming and system design to help the co-design participants imagine, discuss, and visualize the features on the future service.

### Limitations

The most important limitation to consider is the fact that the results are based on only 1 co-design process. By involving 4 researchers (ÅR, CM, PÅ, and CK) in the data collection, and 3 in the data analysis, the researchers’ different preconceptions, viewpoints, and analytic skills were used to achieve high credibility of the results. Nevertheless, some important issues may not have been explored.

Another limitation concerned the data-collection methods. We used video recordings and observations to collect data on the co-design process. When planning the workshops, we discussed the possibility of using 2 cameras to enable simultaneous views of the participants, the interactive board, and the moderator, which is technically complicated. Instead, we connected the camera to a computer and used the Wirecast program to enable simultaneous views. As the workshops proceeded, the participants were clearly more active when using plastic sheets and notes compared with the interactive board. Consequently, we lost the possibility of simultaneous views and had to shift the camera focus between participants, the moderator, and the plastic sheets. The 2 naturalistic observers who were present at the workshops provided an additional overview of the process, which complemented the video recordings in a satisfactory way and contributed to data triangulation.

### Strengths

A unique feature of this study was the use of video recordings and naturalistic observations of the co-design workshops to capture the challenges during the process. Previous empirical studies have used diverse methods to describe and explore the co-design process; for example, surveys [4,12,13], observations and audio-recordings [5], and focus group interviews [27]. We have only been able to identify 1 earlier study that used video recordings to study collaboration during co-design meetings [15]. The use of video recordings provided access to detailed data on the interplay of talk, behavior, and context. It also enabled repeated viewings of the video recordings during analysis and gave the authors access to authentic data. The observation protocols facilitated the identification of the challenges and verified the results from the video-based analysis.

A strength was the use of multiple strategies to secure the credibility of the findings. We used multiple data sources, video recordings, observation protocols, and multiple observers. Researcher triangulation was performed during the different stages of data analysis. By providing a thorough description of the setting, scope of the workshops, methods used, and analysis performed, we intend to make it possible for the reader to assess the transferability of the challenges to similar co-design processes [29]. Our co-design process aimed specifically to develop a mobile Internet service for self-management of PA in RA and included multiple stakeholders in 1 co-design group. The transferability might be unique to processes similar in aim and scope. Nevertheless, the identified challenges at a general level may be valid for other co-design processes attempting to develop and improve health care services and should be evaluated in future services.

### Ethical Considerations

Participatory action research and video-based analysis raises some ethical issues [19]. The use of video-based analysis revealed each participant’s views, preferences, and actions. Therefore, it is of major concern to thoroughly consider how to present the data to retain confidentiality.

### Conclusions

Co-designing the system requirements of a mobile Internet service with multiple stakeholders is a complex and extensive collaborative decision-making process. Considering, valuing, counterbalancing, and integrating different perspectives into agreements and solutions (ie, the merging of participants’ perspectives) were crucial for advancing the process and considered the core challenges of co-design.

This new knowledge of crucial challenges is worth considering when planning and performing future co-design processes on eHealth services that include multiple stakeholders. This study emphasizes that the challenges are crucial for success and should not be omitted but carefully considered and prepared for. Further research is needed to replicate the results in similar and new contexts. Studies on how the participants’ and group characteristics influence the process of merging would also contribute to the field in a significant way. Finally, the inclusion of multiple stakeholders within 1 co-design group is more beneficial than the formation of separate co-design groups for each of the stakeholders with respect to the development and improvement of health care services.

## Abbreviations

EBD: experience-based design
PA: physical activity
RA: rheumatoid arthritis
SRA: Swedish Rheumatism Association
